# Hunters select for behavioral traits in a large carnivore

**DOI:** 10.1038/s41598-019-48853-3

**Published:** 2019-08-26

**Authors:** M. Leclerc, A. Zedrosser, J. E. Swenson, F. Pelletier

**Affiliations:** 1 Canada Research Chair in Evolutionary Demography and Conservation & Centre for Northern Studies, Département de biologie, Université de Sherbrooke, Sherbrooke, J1K2R1 Canada; 2 Faculty of Technology, Natural Sciences and Maritime Sciences, Department of Natural Sciences and Environmental Health, University of South-Eastern Norway, N-3800 Bø i Telemark, Norway; 3 Department of Integrative Biology, Institute of Wildlife Biology and Game Management, University of Natural Resources and Life Sciences, Vienna, Gregor Mendel Str. 33, A - 1180 Vienna, Austria; 4 Faculty of Environmental Sciences and Natural Resource Management, Norwegian University of Life Sciences, PO Box 5003, NO - 1432 Ås Oslo, Norway; 5 Norwegian Institute for Nature Research, NO-7485 Trondheim, Norway

**Keywords:** Behavioural ecology, Behavioural ecology, Evolutionary ecology, Evolutionary ecology

## Abstract

Human harvest can induce selection on life history and morphological traits, leading to ecological and evolutionary responses. Our understanding of harvest-induced selection on behavioral traits is, however, very limited. Here, we assessed whether hunters harvest, consciously or not, individuals with specific behavioral traits. We used long-term, detailed behavioral and survival data of a heavily harvested brown bear (*Ursus arctos*) population in Sweden. We found that hunters harvested male bears that were less active during legal hunting hours and had lower movement rates. Also, hunters harvested male and female bears that used habitats closer to roads. We provide an empirical example that individual behavior can modulate vulnerability to hunting and that hunters could exert a selective pressure on wildlife behaviors. This study increases our understanding of the complex interactions between harvest method, human behavior, and animal behavior that are at play in harvest-induced selection and provides better insight into the full effects of human harvest on wild populations.

## Introduction

Humans are considered an important driver of phenotypic changes in wild populations^1,2^. Phenotypic changes, including both plastic and genetic responses, have been documented in the wild, and meta-analyses suggest that trait changes occur at a faster rate in anthropogenic landscapes, particularly when humans act as predators and harvest wild populations^3,4^. Mortality due to human harvest typically differs from natural mortality, because in most harvest systems humans often harvest adult individuals, which usually show high survival when not harvested^5^. Human harvest is also often nonrandom and commonly harvest specific phenotypes^6,7^, such as in size-selective fisheries or trophy hunting, which might result in harvest-induced selection and evolution^8^.

Empirical studies have documented evolutionary changes in morphological and life-history traits in response to size-selective harvest^9,10^. Trophy hunting of male bighorn sheep (*Ovis canadensis*) has been suggested to select and cause evolution towards smaller horn size^10^. There is, however, an ongoing debate whether hunting may induce contemporary evolution in terrestrial ecosystems^11,12^. On the other hand, harvest-induced selection and evolution in fisheries is well documented and accepted. For instance, harvest was shown to reduce annual body growth and induce maturation at a lower age and smaller size in several harvested fish populations^13,14^. Biro and Post^15^ showed that size-selective harvesting also may induce selection on behavioral traits. In a whole-lake experiment, fast-growing and bold rainbow trout (*Oncorhynchus mykiss*) were more likely to be caught in gillnets compared to slow-growing and shy individuals^15^. This result highlights that harvest-induced selection on behaviors might be widespread, given the important use of size-selective harvest methods in fisheries. Empirical tests for such predictions from wild populations, however, remains scarce, particularly for terrestrial ecosystems^16^.

The goal of this study was to investigate if hunters harvest, consciously or not, individuals with specific behavioral traits and thereby induce selection on behaviors in a heavily harvested terrestrial carnivore population^17,18^. We used detailed behavioral information derived from GPS relocations and activity sensors from individually marked brown bears (*Ursus arctos*) in a Swedish population. Brown bear hunting in Sweden takes place in the fall and hunting quotas are set at the county level. As long as the quota has not been filled, there is no limit on the number of bears that an individual hunter can harvest^19^. During the study period (2003–2016), baiting was not allowed, and most hunters used unleashed baying dogs (maximum 2 dogs) to find and track bears^19^. Dogs were released from the leash when they picked up a bear’s scent. Hunters then followed the dogs and tried to harvest the bear.

Here, we assessed hypotheses that explored the behavioral differences of harvested bears and those that survived the hunting season. First, we tested whether bears that survive more hunting seasons display behaviors that reduce their probability of encountering hunters, and we predicted that bears that survive more hunting seasons would use habitats farther from roads. We also explored if hunters harvest bears that are more active and potentially leave more tracks and scent that can be picked up by a hunting dog. Based on this hypothesis, we expected that bears that survive more hunting seasons would have lower rates of movement and be less active during legal hunting hours. Alternatively, bears that are more active may escape approaching dogs and hunters more easily than inactive bears. Based on this alternative hypothesis, we predicted that bears that survive more hunting seasons would have higher rates of movement and be more active during legal hunting hours.

## Results

**Figure 1:**
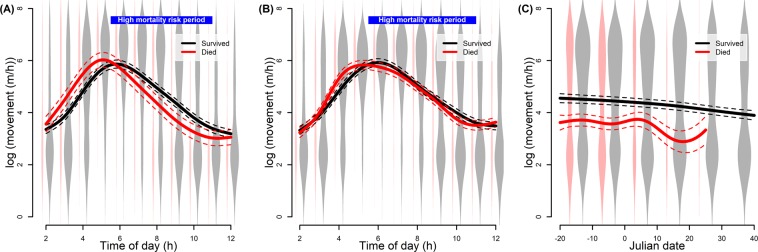
Predicted (solid line) variation and 95% CIs (dashed lines) in rates of movement (log-transformed) for the most parsimonious model tested for male and female brown bears in Sweden (2003–2016). Shown are the effect of the time of day (panel A,B) and Julian date (panel C) for males (panel A,C) and females (panel B). Julian date 0 represents the start of the hunting season. Violin plots in the background represent the distribution of raw data.

The database included 32,849 rates of movement (distance travelled in 1 h) from 41 males (87 bear-years) and 35,821 rates of movement from 37 females (92 bear-years) during 2003 to 2016. For male rates of movement, the most parsimonious model (Table S1) included a negative effect of age (Figure S1, estimated degree of freedom [edf] = 2.255, p-value < 0.001), the time of day by hunting season fate (Fig. 1A, p-value < 0.001), and Julian date by hunting season fate (Fig. 1C, p-value < 0.001). Predictions suggested that, at 04:00, males that died during a hunting season moved twice as fast as males that survived a hunting season (245 m/h vs 125 m/h), although that trend was reversed at 09:00 (Fig. 1A; died = 42 m/h and survived = 81 m/h). The male model’s marginal *R*^2^ was 23.3%. The most parsimonious model (Table S1) for female rates of movement included a negative effect of age (Figure S2a, edf = 1.891, p-value < 0.001) and Julian date (Figure S2b, edf = 3.347, p-value < 0.001), and time of day by hunting season fate (Fig. 1B, p-value < 0.001). Predictions suggested that, at 04:00, females that died during a hunting season moved significantly more than females that survived a hunting season (206 m/h vs 126 m/h), but there were no important differences in rates of movement during the high mortality risk period (Fig. 1B). The female model’s marginal *R*^2^ was 24.4%. We found that rates of movement were significantly repeatable across individuals for males (*R* = 0.123, p-value < 0.001) and females (*R* = 0.118, p-value < 0.001).

**Figure 2:**
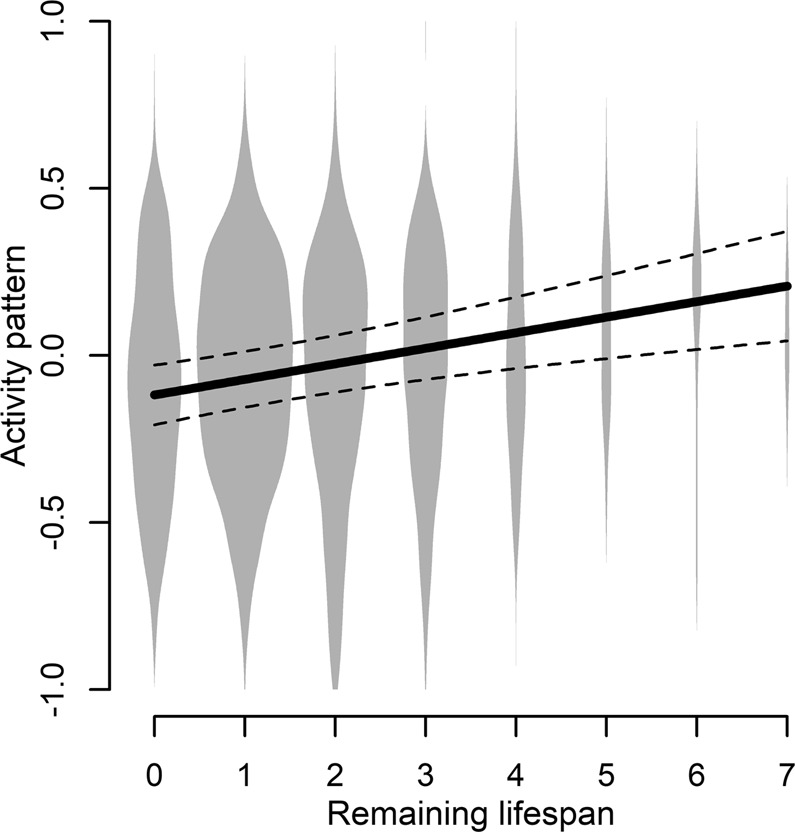
Predicted (solid line) variation and 95% CIs (dashed lines) in male brown bear activity patterns from the most parsimonious model tested. Shown is the relationship between activity pattern and the remaining lifespan. Values of -1 and 1 indicates that all activity occurred during nonhunting and hunting hours, respectively. Violin plots in the background represent the distribution of raw data.

The database for activity pattern (obtained from dual-axis motion activity sensors) included 3,356 daily values from 38 males (82 bear-years) and 4,145 daily values from 37 females (92 bear-years) during 2003 to 2016. The most parsimonious model (Table S2) for male activity patterns included a negative effect of age (Figure S3a, edf = 3.507, p-value < 0.001), a positive effect of the remaining lifespan (Fig. 2, edf = 1, p-value < 0.001), and the variable Julian date (Figure S3b, edf = 5.038, p-value < 0.001). The male model’s marginal *R*^2^ was 10.5%. For female activity patterns, the most parsimonious model (Table S2) only included age (Figure S4a, edf = 3.039, p-value = 0.016) and Julian date (Figure S4b, edf = 5.165, p-value < 0.001). The female model’s marginal *R*^2^ was 4.1%. Activity patterns were significantly repeatable across individuals for males (*R* = 0.284, p-value < 0.001) and females (*R* = 0.369, p-value < 0.001).

**Figure 3:**
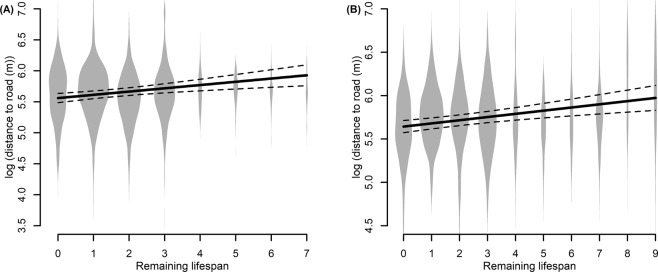
Predicted (solid line) variation and 95% CIs (dashed lines) in the daily mean distance to roads in function of the remaining lifespan from the most parsimonious model tested for male (panel A) and female (panel B) brown bears in Sweden (2003–2016). Violin plots in the background represent the distribution of raw data.

The mean daily distance to a road included 2,566 and 3,711 values from 31 males (69 bear-years) and 35 females (81 bear-years), respectively, during 2003 to 2016. For males’ mean daily distance to a road, the most parsimonious model (Table S3) included a negative effect of road density (Table 1, β = -0.706, p-value < 0.001), Julian date (Table 1, β = -0.001, p-value = 0.026), and a positive effect of the remaining lifespan (Fig. 3A, β = 0.053, p-value = 0.001). Age (β = 0.007, p-value = 0.355) also was included in the most parsimonious model, but its 95% confidence intervals included 0 (Table 1). The male model’s marginal *R*^2^ was 20.5%. For females’ mean daily distance to a road, the most parsimonious model (Table S3) included a negative effect of road density (Table 1, β = -0.406, p-value = 0.005) and Julian date (Table 1, β = -0.002, p-value < 0.001), and a positive effect of the remaining lifespan (Fig. 3B, β = 0.037, p-value < 0.001). Age (β = -0.001, p-value = 0.844) also was included in the most parsimonious model, but its confidence intervals included 0 (Table 1). The female model’s marginal *R*^2^ was 27.9%. Mean daily distances to a road were significantly repeatable across individuals for males (*R* = 0.225, p-value < 0.001) and females (*R* = 0.281, p-value < 0.001).

**Table 1:** Coefficients (β) and their 95% confidence intervals (C.I.) of the most parsimonious model that explained variation in the daily mean distance to roads of male and female brown bears in Sweden (2003–2016).

Variable	Male (*n* = 31)			Female (*n* = 35)		
	β	Lower C.I.	Upper C.I.	β	Lower C.I.	Upper C.I.
Intercept	**6.265**	**5.871**	**6.658**	**6.087**	**5.779**	**6.396**
Home range road density*	−**0.706**	−**1.089**	−**0.323**	−**0.406**	−**0.680**	−**0.133**
Bear age	0.007	−0.008	0.021	−0.001	−0.014	0.012
Julian date**	−**0.001**	−**0.003**	−**0.000**	−**0.002**	−**0.003**	−**0.001**
Remaining lifespan	**0.053**	**0.023**	**0.082**	**0.037**	**0.018**	**0.055**

*km of roads/km^2^; **where 0 = 21 August, i.e., the start of the hunting season.

Coefficients with C.I. that do not overlap with 0 are shown in bold.

## Discussion

Using a long-term behavioral dataset from a harvested population in a terrestrial ecosystem, we tested if hunters harvested individuals with specific behavioral traits in a heavily harvested terrestrial carnivore population. Using detailed individual-based behavioral information from brown bears in Sweden, we showed that both males and females with greater remaining lifespan were found farther away from roads than individuals with lower remaining lifespan. We also showed that males with greater remaining lifespan were more active during legal hunting hours compered to males with lower remaining lifespan. Finally, we showed that males and females that were harvested in a given year expressed different rates of movement throughout the day than males and females that were not harvested. Differences in behaviors were repeatable across individuals for both males and females. Therefore, we provide evidence that hunting mortality is correlated to bear behaviors and that hunting likely exerts a selective pressure on the behavior of Scandinavian brown bears.

We showed that bears with a greater remaining lifespan were found farther from roads (Fig. 3). This result can likely be explained by hunters actively using the road network to move across the landscape and access hunting posts, thereby increasing the probability of detecting a bear close to roads, which results in bears being harvested closer to roads. A previous study in the same bear population found higher mortality risk in habitats closer to roads^20^. A similar pattern also was found in Canada, where hunters preferentially killed brown bears and elk (*Cervus elaphus*) that were closer to roads^21,22^. We also found that male bears that are more active during legal hunting hours and have higher rates of movement may better avoid being killed by hunters. We hypothesized that male bears that were usually more active and had higher rates of movement during the high mortality risk period may initiate the escape from hunting dogs more quickly, which may increase their survival probability, compared to bears that were more inactive and stationary during the high mortality risk period^23^. Indeed, hunters and their dogs are not always successful even when finding recent bear tracks, as bears are able to escape by moving away from the dogs and hunters^24^. Although the probability of a bear escaping when chased by dogs and hunters is unknown in our study population, it has been shown that American black bears (*Ursus americanus*) can escape and survive 75–86% of the times they were chased by hunting dogs^24^. Higher rates of movement, however, may not always translate into higher probability of escaping hunters. For instance, Ciuti *et al*.^21^ showed that elk that died from hunting had higher rates of movement compared to elk that survived. In their study systems, elk with higher rates of movement might have been more easily detected by hunters searching for them using binoculars and spotting scopes^21^. Therefore, different harvest regulations and hunting strategies used by humans might influence the direction of the selective pressure caused by harvest.

Contrary to males, we did not detect hunter selection for females’ activity patterns or rates of movement during the high mortality risk period. We suggest that the Swedish hunting regulations might reduce the strength of the selective pressure on females. Indeed, family groups are legally protected from hunting and offspring stay with their mother for 1.5 years or 2.5 years^7^. Therefore, female bears can usually be legally harvested only every 2 or 3 year, in contrast to males, which can be legally harvested every year.

We found evidence that hunting may disrupt the activity patterns of bears^25^. Bears become more day active during hyperphagia (time period during summer and fall when bears try to accumulate adipose tissue for hibernation), however, this trend stopped and was even reversed with the onset of the hunting season (Figure S3–S4). Similar patterns were observed by Ordiz *et al*.^25^, who showed that bears are more active during the night after the onset of the hunting season. With advancing age, bears also tended to be more night active and had lower rates of movement (Figure S1–S4). Such behavioral plasticity within and among hunting seasons might not be adaptive, as bears that had greater remaining lifespan or survived a hunting season were more day active and had higher rates of movement, respectively.

Predicting the consequences of harvest-induced selection on behavioral traits in harvested populations can be complex, but a recent framework in fisheries suggest that passive gear should harvest bolder individuals and lead to a timidity syndrome in the harvested population, whereas active gear should harvest shyer individuals and lead to a boldness syndrome^26,27^. This framework accounts for the interplay between the harvest method and animal behavior, but it may allow broader predictions if it also accounts for human behavior and harvested species ecology^28,29^. For instance, in terrestrial ecosystems, we known human access is an important driver explaining distribution of hunting mortalities^20,30^. Therefore, we proposed that, at a larger spatial scale, behaviors that increases the encounter probability between an animal and a hunter or a fishing gear should be selected against by harvesting. At a smaller spatial scale, once an animal is in the vicinity of a hunter or of fishing gear, bolder individuals will be more vulnerable to passive harvest methods and shyer individuals will be more vulnerable to active harvest methods^16,26,27,31^. Also, one must carefully integrate information about the harvested species’ ecology and behavior. For instance, in terrestrial ecosystems, rates of movement may influence both the probability of being detected by dogs or hunters and the probability to escape hunting dogs and hunters. In our population, we know that bears sometimes do escape hunters and their dogs. Other species, however, like the cougar (*Puma concolor*), could be more likely to climb a tree when chased by dogs, which increases the probability of being harvested^24^. Therefore, we propose that rates of movement can be selected for (when detection probability < probability of escaping dogs) or against (when detection probability > probability of escaping dogs), depending on a species’ ecology. More studies in both marine and terrestrial ecosystems are needed to test this updated framework and better understand the complex interactions at play in behavioral harvest-induced selection.

To test predictions of harvest-induced selection on behavioral traits with the proposed framework, the behaviors from individuals must be correctly identified as “bold” or “shy”. However, this is not a trivial exercise. For instance, repeatable individual variation in habitat selection of bogs has been shown in our population^32^, but it would be tenuous to infer that one bear is bolder than another based on this behavior. To make comparisons between studies more feasible and to develop more general predictions, we propose to use behaviors shown or predicted to be under selection, rather than an imprecise secondary term such as “boldness”^33,34^.

In this study, we explored behavioral differences between bears that died and bears that survived hunting seasons. We showed correlations between two proxies of bear survival (hunting season fate and remaining lifespan) and bear behaviors during hunting season, suggesting that hunters exert a selective pressure on behavioral traits. Such selective pressure can have important ecological and evolutionary implications^35–37^. For instance, changes in behavioral traits caused by harvest-induced selection on male rates of movement could influence the occurrence of sexually selected infanticide in our population^38–40^, which is known to affect population dynamics^18,41,42^. Harvest can also cause ecological effects on other species than the one harvested^35,36^. For instance, the brown bear is a top predator and a great seed disperser, thus behavioral changes in bears could influence both top-down and bottom-up processes^43–45^. Ecological effects of harvest-induced traits changes have not been quantified in our population, but have been observed in other systems^35,36^. To document evolutionary effects of harvest, one must show that traits are variable between individuals (repeatable), that the traits are under selection, and that the traits are heritable. Here, we did find repeatable individual differences in behaviors^32,46^ that were associated with fitness proxies. It is unknown, however, if those behavioral traits are heritable. Nevertheless, meta-analyses suggest that behaviors usually have a higher heritability than life history traits^47,48^. Therefore, behavioral harvest-induced evolution could occur in our brown bear population.

We provide empirical evidence that individual behavior can modulate vulnerability to hunting. Our results, when compared to other studies on harvest-induced selection, suggest that harvest regulations and human behavior may influence the direction of selection imposed by harvest. By better integrating the marine and terrestrial literature on harvest-induced selection and by developing collaborations, we should be able to better understand and quantify the full effects of human harvest on wild populations.

## Methods

### Study area

The study area was located in southcentral Sweden (61°N, 15°E) and is comprised of bogs, lakes, and intensively managed coniferous forest stands of variable ages. The dominant tree species were Norway spruce (*Picea abies*), Scots pine (*Pinus sylvestris*), and birches (*Betula* spp.). Elevations ranged between 150 and 1000 m asl. The study area is intersected with a dense network of forest roads. Human population density is among the lowest in the European brown bear range, with humans concentrated in villages in the northern and southern parts of the study area. Small settlements and isolated houses are scattered throughout the area. See Martin *et al*.^49^ for further information about the study area.

We captured 79 brown bears (41 males and 38 females) from a helicopter (2003–2016) using a remote drug delivery system (Dan-Inject, Børkop, Denmark). We extracted a vestigial first premolar for age determination from each individual not captured as a yearling with its mother^50^. See Fahlman *et al*.^51^ for further details on capture and handling. We equipped bears with GPS collars (GPS Plus; Vectronic Aerospace, Berlin, Germany) programed to relocate a bear every hour. Collars also included dual-axis motion activity sensors that measured acceleration in two orthogonal directions. Measures of acceleration were averaged every 5 minutes and stored in the collar. All captured bears were part of the Scandinavian Brown Bear Research Project and all experiments, captures and handling were performed in accordance with relevant guidelines and regulations and were approved by the appropriate authority and ethical committee (Naturvårdsverket and Djuretiska nämden i Uppsala, Sweden).

Brown bear hunting in Sweden starts on 21 August and the hunting season lasts until the scheduled season end date (15 October in the study area) or until the harvest quota is reached (whichever comes first). Legal hunting hours (hereafter ‘hunting hours’) in Sweden last from one hour before sunrise until two hours before sunset. Members of family groups, i.e. females accompanied by offspring of any age, are protected and cannot be legally harvested^52^. Trophy hunting targeting large bears is rare, but financially motivated guided hunts have increased in Sweden in recent years^19,52^.

### Data handling

We used information from GPS collars and dual-axis motion activity sensors from individual bears in years when they could be legally harvested (i.e., were not in a family group). For each bear, we used data from 1 August to 30 September each year (hereafter “bear-year”) to cover the period before and after the start of the hunting season. We screened GPS relocation data and removed GPS fixes with dilution of precision values >10 to increase spatial accuracy (~2 to 4% of bear GPS relocations). We then used these GPS data to quantify two behavioral traits that may affect vulnerability to hunting, i.e., rate of movement and distance to closest road. Rate of movement was calculated as the Euclidian distance traveled by a bear during a 1-hour interval using the “adehabitatLT” package in R 3.4.4^53^. Proximity to road was calculated with the “rgeos” package and defined as the Euclidian distance between GPS relocations of bears and the closest road using maps of the Swedish National Road Database from the Swedish Transport Administration (© Trafikverket). We averaged the daily Euclidian distances to the closest road for easier model convergence and we calculated road density within each “bear-year” home range.

The third behavioral trait, activity pattern, was quantified using data from dual-axis motion activity sensors. For each individual, we calculated a daily index of activity during hunting hours, corrected for daylight changes, following Hoogenboom *et al*.^54^:

$$\frac{\frac{S{A}_{h}}{{D}_{h}}-\frac{S{A}_{nh}}{{D}_{nh}}}{\frac{S{A}_{h}}{{D}_{h}}+\frac{S{A}_{nh}}{{D}_{nh}}}$$

where SAh and SAnh are the sum of activity values during hunting hours and nonhunting hours, respectively, and where Dh and Dnh are the duration of hunting hours and nonhunting hours, respectively. This index ranges between −1 and 1, where −1 represents a bear that is only active during nonhunting hours, and 1 represents a bear that is only active during hunting hours.

### Statistical analysis

Because we wanted to investigate the direction of harvest-induced selection on behaviors, we used behavioral information from bears that had died from hunting (88.3% of bears that died during the study period; other mortality causes were unknown (8.5%), management (1.2%), vehicle collision (1.2%), and capture (0.8%)). We have modeled behavior (dependent variable), not survival, because modeling behaviors allowed us to include control covariates, such as age and Julian date, which are known to influence behavioral traits [as done in Ciuti *et al*.^21^]. In addition to control covariates, we included one of two variables describing bear longevity to show differences between bears that died and those that survive. The first variable was hunting season fate, a dummy variable describing whether the bear was harvested or not during the hunting season. The second variable was initially age at death (lifespan), but it was highly correlated (r > 0.9) with age. We therefore used the remaining lifespan, i.e. the interval between the year of behavioral data collection and the year the bear was harvested. The remaining lifespan was not collinear with age (*r* < 0.35) and all Variation Inflation Factors (VIF) <3 when both variables were included in the same model. We ran candidate models for each sex separately.

To model rates of movement (dependent variable), we used general additive mixed models (“mgcv” package) of the Gaussian family, which allow flexible specification of the relationships, instead of being forced to be linear or quadratic^55^. Due to convergence issues and to avoid extensive computational time (>months), we focused on modeling the behavior of individual bears from 02:00 to 12:00, which included the period of the day with the highest hunting mortality risk for brown bears in our study area (around 07:00, see Fig. 1 in Hertel *et al*.^56^). We tested a set of candidate models constructed hierarchically (Table S1), based on the following independent variables: hour of day, Julian date, age of the bear, hunting season fate, and the remaining lifespan. We log-transformed movement data to fulfill assumptions of normality of residuals and homogeneity of variance. We also used general additive mixed models to model activity patterns (dependent variable) and, as for modeling rates of movement, we tested a set of candidate models constructed hierarchically (Table S2), based on the following independent variables: Julian date, age of the bear, hunting season fate, and the remaining lifespan. We modeled the mean daily distance to the closest road (dependent variable) with linear mixed models (“nlme” package) instead of general additive mixed models, because all relationships with the independent variables were linear. We again tested a set of candidate models constructed hierarchically (Table S3), based on the following independent variables: Julian date, road density in the home range, age of the bear, hunting season fate, and the remaining lifespan. The mean daily distance to the closest road was log-transformed to fulfill statistical assumptions. All candidate models tested included “bear-year” nested in “Bear ID” as random intercepts and an AR1 function controlling for temporal autocorrelation. All candidate models were ranked using AICc^57^. We also calculated the adjusted (i.e. conditional on the model’s fixed effects) behavioral repeatability (*R*) of the most parsimonious model using:

$$R=\frac{{S}_{BearID}^{2}}{{S}_{BearID}^{2}+{S}_{bearyear}^{2}+{S}_{residual}^{2}}$$

where ${S}_{BearID}^{2}$ is the variance among individuals, ${S}_{bearyear}^{2}$ is the variance across years within individuals, and ${S}_{residual}^{2}$ is the residual variance of the model, i.e. the variance within individuals within a year. Repeatability varies between 0 and 1, where 0 suggests that there is no variation among individuals, and 1 suggests that all variation observed is due to individual differences. All spatial and statistical analyses were performed in R 3.4.4^53^. This manuscript is part of M. Leclerc PhD thesis^58^.

## Supplementary information

Supplementary information

R-script

Dataset

## Data Availability

All data and code are available in supplementary materials.
