# Supplementary material for: Hunters select for behavioral traits in a large carnivore
Source: Sci Rep. 2019 Aug 26;9:12371. doi: 10.1038/s41598-019-48853-3 (PMC6710287; doi:10.1038/s41598-019-48853-3)

Supplementary Information

Hunters select for behavioral traits in a large carnivore

M. Leclerc, A. Zedrosser, J.E. Swenson, F. Pelletier

Correspondence to: Martin.Leclerc2@USherbrooke.ca

Table S1. Candidate models tested to explain variation in rates of movement.

Table S2. Candidate models tested to explain variation in activity pattern.

Table S3. Candidate models tested to explain variation in the daily mean distance to roads.

Figure S1. Predictions of male brown bear rates of movement.

Figure S2. Predictions of female brown bear rates of movement.

Figure S3. Predictions of male brown bear activity pattern.

Figure S4. Predictions of female brown bear activity pattern.

Table S1. Candidate models tested to explain variation in rates of movement (log-transformed) of brown bears in Sweden (2003-2016). All models included “bear-year” nested in “Bear ID” as random intercepts and an AR1 temporal autocorrelation function. Models are listed with their variable, log likelihood (LL), and difference in AICc to the most parsimonious model (ΔAICc).

		Male (*n*=41)		Female (*n*=37)	
Model	Variable*	LL	ΔAICc	LL	ΔAICc
1	None	-65091.0	5445.8	-68781.3	6493.3
2	Age	-65080.9	5429.6	-68774.5	6483.7
3	Age + Time of day	-62506.9	285.6	-65690.7	320.2
4	Age + Time of day by hunting season fate	-62423.5	122.8	-65668.2	279.1
5	Age + Julian date	-65038.4	5348.7	-68676.8	6292.5
6	Age + Julian date by hunting season fate	Did not	converge	-68675.4	6293.5
7	Age + Remaining lifespan	-65079.1	5429.9	-68772.7	6484.2
8	Age + Time of day + Julian date	-62444.9	165.5	-65549.9	42.4
9	Age + Time of day + Julian date by hunting season fate	-62443.0	165.9	-65548.6	44.0
10	Age + Time of day + Remaining lifespan	-62504.8	285.4	-65690.1	322.9
11	Age + Time of day by hunting season fate + Julian date	-62360.3	0.3	**-65526.6**	**0**
12	Age + Time of day by hunting season fate + Julian date by hunting season fate	**-62358.1**	**0**	-65525.2	1.1
14	Age + Time of day + Julian date + Remaining lifespan	-62437.7	155.2	-65546.5	39.8

* All variables, except hunting season fate (died/survive), were fitted with smoothing splines, which allows flexible specification of the relationships, instead of forcing them to be linear, quadratic, or cubic.

Table S2. Candidate models tested to explain variation in activity pattern of brown bears in Sweden (2003-2015). All models included “bear-year” nested in “Bear ID” as random intercepts and an AR1 temporal autocorrelation function. Models are listed with their variable, log likelihood (LL), and difference in AICc to the most parsimonious model (ΔAICc).

		Male (*n*=38)		Female (*n*=37)	
Model	Variable*	LL	ΔAICc	LL	ΔAIC
1	None	-33.3	53.3	524.0	61.1
2	Age	-24.3	39.3	526.1	60.9
3	Age + Julian date	-7.1	9.0	**558.5**	**0**
4	Age + Julian date by hunting season fate	-6.4	11.6	557.4	6.3
5	Age + Remaining lifespan	-18.1	31.1	527.0	63.1
6	Age + Julian date + Remaining lifespan	**-0.6**	**0**	559.4	2.3

* All variables, except hunting season fate (died/survive), were fitted with smoothing splines, which allows flexible specification of the relationships, instead of forcing them to be linear, quadratic, or cubic.

Table S3. Candidate models tested to explain variation in the daily mean distance to roads in brown bears in Sweden (2003-2016). All models included “bear-year” nested in “Bear ID” as random intercepts and an AR1 temporal autocorrelation function. Models are listed with their variable, log likelihood (LL), and difference in AICc to the most parsimonious model (ΔAICc).

		Male (*n*=31)		Female (*n*=35)	
Model	Variable*	LL	ΔAICc	LL	ΔAICc
1	None	-1097.1	13.3	-1358.2	37.69
2	Home range road density + Age	-1094.2	11.5	-1353.1	31.36
3	Model 2 + Julian date	-1092.5	10.3	-1343.0	13.19
4	Model 2+ Julian date + Hunting season fate + Julian date × Hunting season fate	-1089.1	7.4	-1337.5	6.21
5	Model 2 + Remaining lifespan	-1088.9	3.0	-1346.5	20.3
6	Model 2 + Julian date + Remaining lifespan	**-1086.4**	**0.0**	**-1335.4**	**0**

Figure S1. Predicted variation in rates of movement (log-transformed) for the most parsimonious model tested for male (*n* = 41 bears, 32,849 movements) brown bears in Sweden. Shown are the effect of bear age (estimated degree of freedom = 2.255, p-value < 0.001).

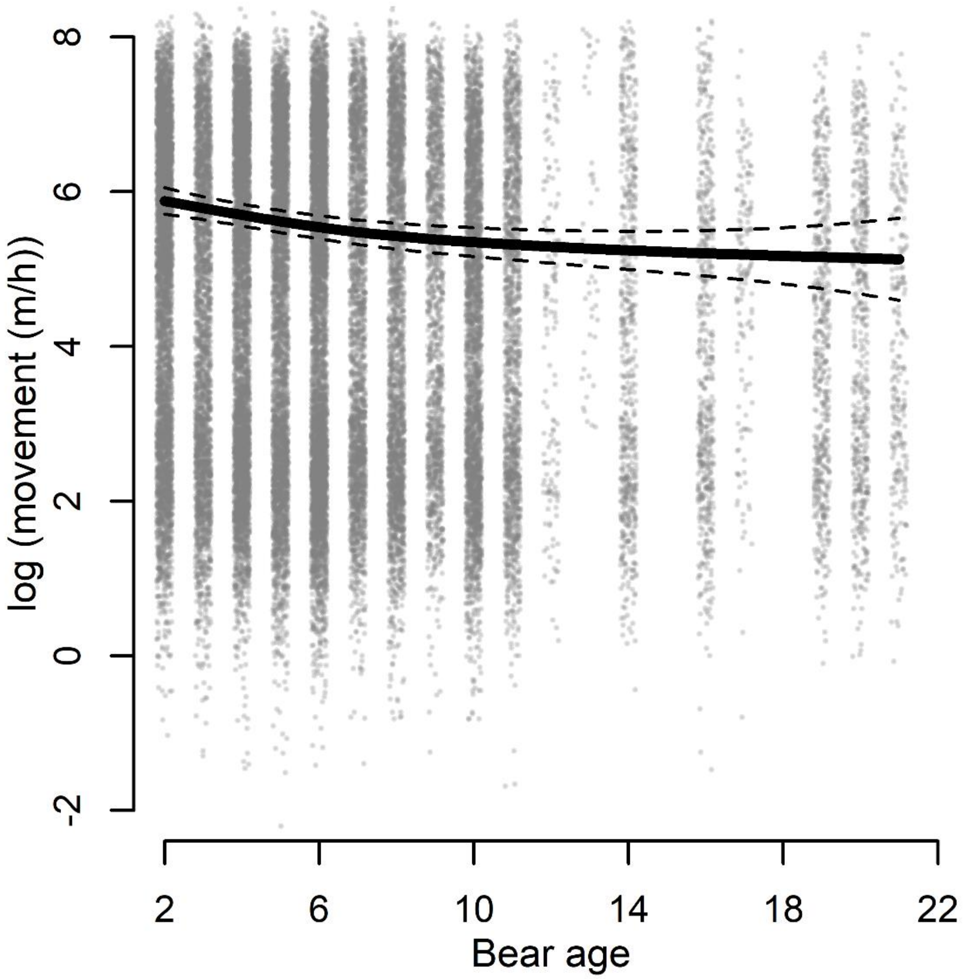

Figure S2. Predicted variation in rates of movement (log-transformed) for the most parsimonious model tested for female (*n* = 37 bears, 35,821 movements) brown bears in Sweden. Shown are the effect of bear age (panel a, estimated degree of freedom = 1.891, p-value < 0.001) and Julian date (panel b, estimated degree of freedom = 3.347, p-value < 0.001) where 0 represents the start of the hunting season.

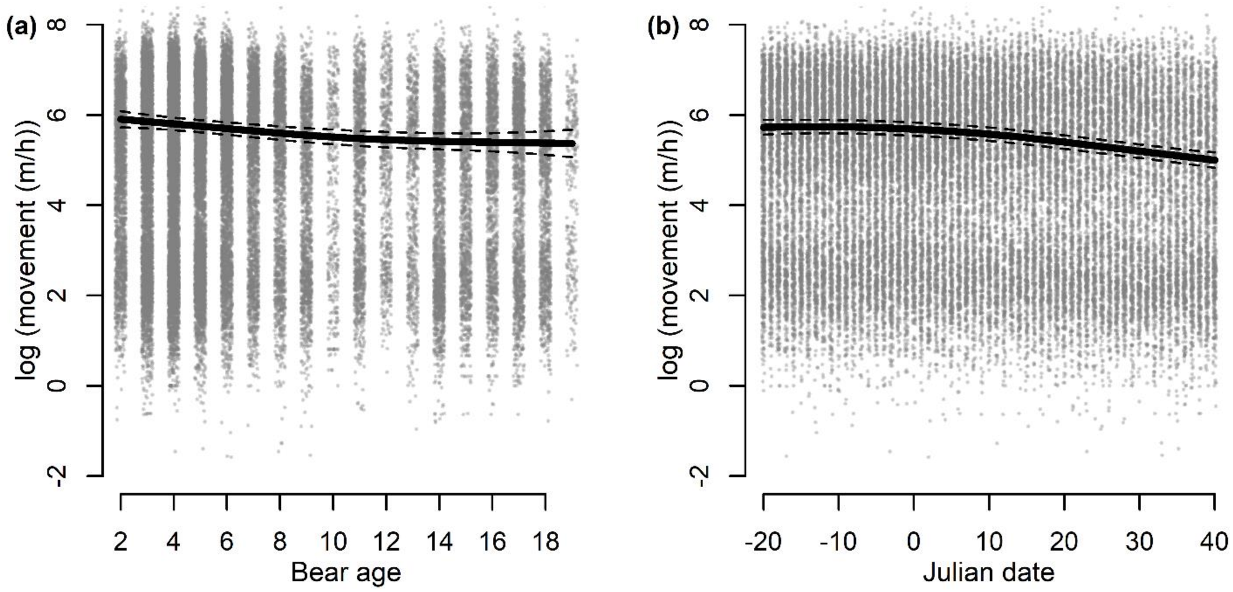

Figure S3. Predicted variation of male brown bear (*n* = 38 bears, 3,356 daily values) activity patterns from the most parsimonious model tested. Shown is the relationship between activity pattern and bear age (panel a, estimated degree of freedom = 3.507, p-value < 0.001) and Julian date (panel b, estimated degree of freedom = 5.038, p-value < 0.001), where 0 represents the start of the hunting season. Activity pattern values of -1 and 1 indicate that all activity occurred during nonhunting and hunting hours, respectively.

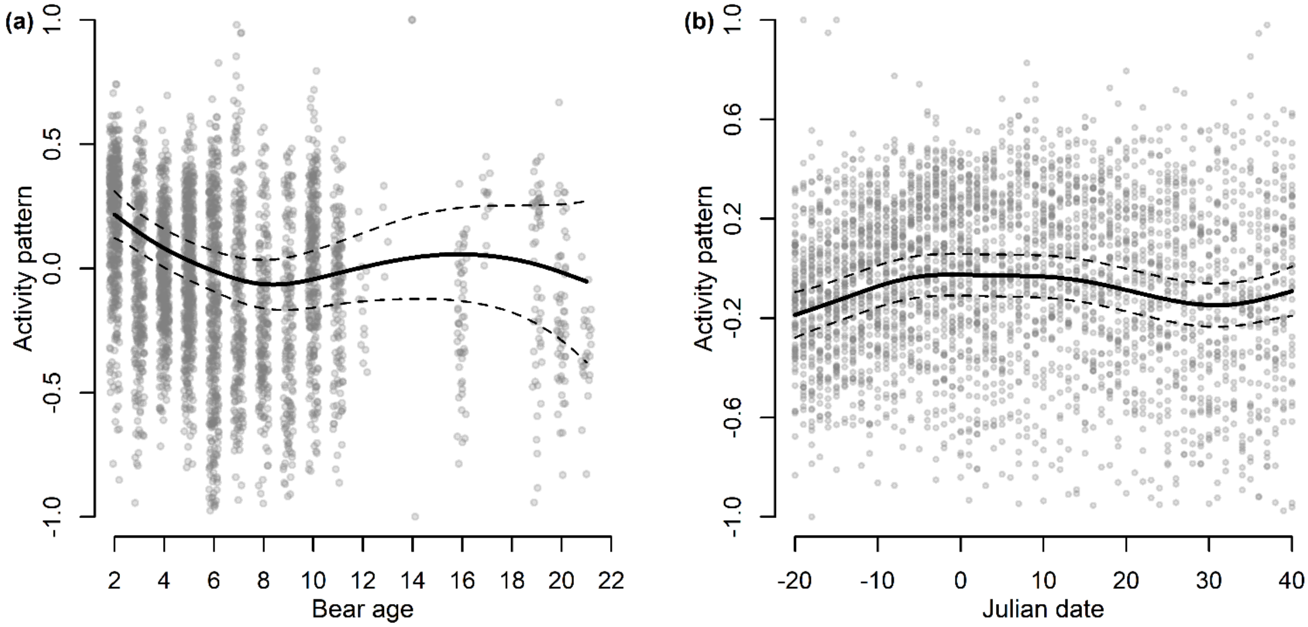

Figure S4. Predicted variation of female brown bear (*n* = 37 bears, 4,145 daily values) activity patterns from the most parsimonious model tested. Shown is the relationship between activity pattern and bear age (panel a, estimated degree of freedom = 3.039, p-value = 0.016) and Julian date (panel b, estimated degree of freedom = 5.165, p-value < 0.001), where 0 represents the start of the hunting season. Activity pattern values of -1 and 1 indicate that all activity occurred during nonhunting and hunting hours, respectively.

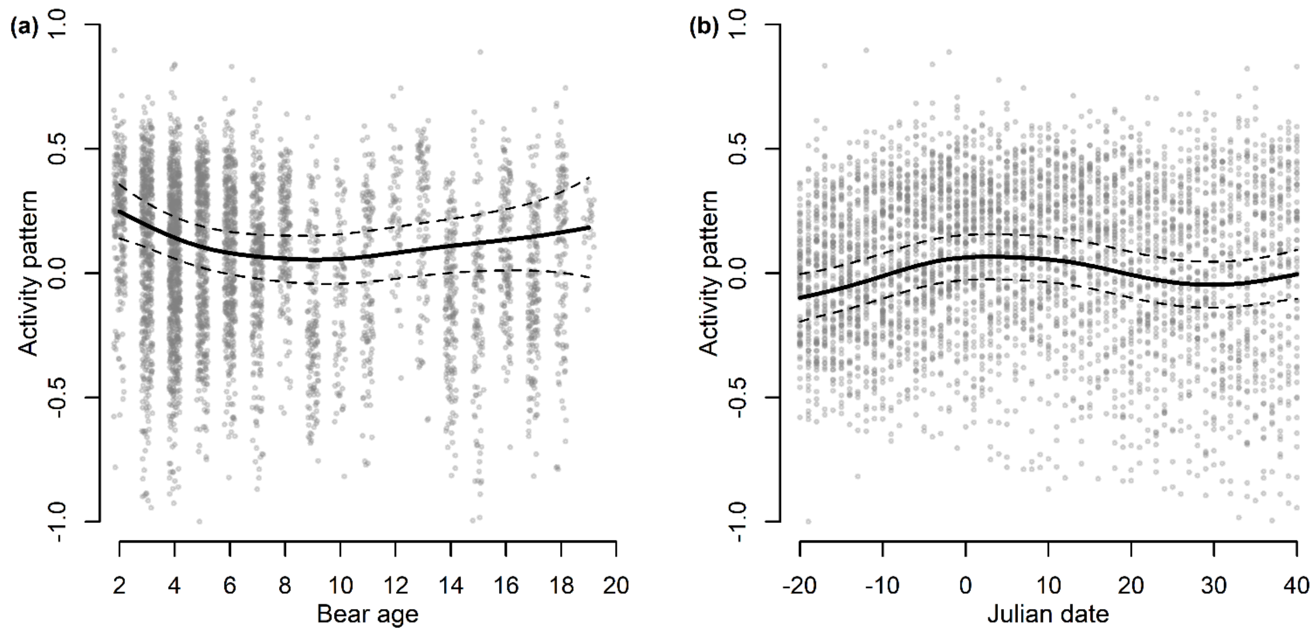

Supplement: Supplementary file 1 — Supplementary information [file 41598_2019_48853_MOESM1_ESM.pdf]
